# Phylogenetic and Evolutionary Genomic Analysis of *Listeria monocytogenes* Clinical Strains in the Framework of Foodborne Listeriosis Risk Assessment

**DOI:** 10.3389/fmicb.2022.816880

**Published:** 2022-04-01

**Authors:** Antonietta Gattuso, Eleonora Cella, Silvia Fillo, Marco Francesco Ortoffi, Silvia Angeletti, Massimo Ciccozzi, Dario De Medici, Florigio Lista, Alfonsina Fiore

**Affiliations:** ^1^Dipartimento di Sicurezza Alimentare, Nutrizione e Sanità Pubblica Veterinaria, Istituto Superiore di Sanità, Rome, Italy; ^2^Unità di Statistica Medica ed Epidemiologia Molecolare, Università Campus Bio-Medico di Roma, Rome, Italy; ^3^Scientific Department, Army Medical Center, Rome, Italy

**Keywords:** *Listeria monocytogenes*, listeriosis, whole-genome sequencing (WGS), clonal complexes (CCs), sequence types (STs), phylogenetic analysis, lineage, hazard characterization

## Abstract

*Listeria monocytogenes* is one of the most important foodborne pathogens responsible for listeriosis, a severe disease with symptoms ranging from septicemia, meningoencephalitis, and abortion. Given the strong impact of listeriosis on human health and the difficulty of controlling *L. monocytogenes* along the food production chain, listeriosis has become a priority subjected to molecular surveillance in European Union/European Economic Area since 2007. From 2018, surveillance is based on whole-genome sequence using the core genome multilocus sequence type. The complete sequences of 132 clinical strains were used to define the evolutionary relatedness among subtypes of *L. monocytogenes* isolated in Italy from 2010 to 2016, allowing the identification of clades and/or clusters associated with outbreaks or sporadic cases of listeriosis. All the strains analyzed are clustered in lineages I and II, and the majority of the strains were classified as lineage II. A probable epidemic entrance in different years for every clade and cluster from each different region was defined. The persistence of the same specific clonal complexes of *L. monocytogenes* has been found over long periods; this may be related to the fact that some strains are able to survive better than others in a food production environment. Phylogenic studies, using whole-genome sequence data, are able to identify the emergence of highly persistent pathogenic variants, contributing to improving the hazard characterization of *L. monocytogenes*.

## Introduction

*Listeria monocytogenes*, one of the most important foodborne pathogens, is the causative agent of invasive listeriosis that typically presents as sepsis or meningoencephalitis in the elderly (>65 years) and in people with chronic illnesses and undergoing immunosuppression (de Noordhout et al., 2014). Infections during pregnancy can cause fever and other non-specific symptoms in the mother with severe outcomes such as fetal loss, premature labor, neonatal illness, and death (Lamont et al., 2011). Listeriosis can have a relatively long incubation period, many cases are considered sporadic, and detected outbreaks usually involve a small number of patients, so most reported cases of listeriosis are difficult to link to a specific food product or food business operator (Van Walle et al., 2018). *L. monocytogenes* is able to form biofilms to grow at refrigeration temperature, tolerate high salt and nitrite concentrations, and resist disinfectants (Lundén et al., 2003). These properties contribute to its ability to persist and multiply in the food-processing environment and make it difficult to control. *L. monocytogenes* demonstrated a high genome-level of diversity, forming four evolutionary lineages (I–IV; Ragon et al., 2008); the majority of human illnesses were caused by strains belonging to lineages II and I. Given the strong impact of listeriosis on human health and the difficulty of controlling the pathogen along the food production chain, in 2007, the European Center for Diseases Prevention and Control (ECDC) identified within the food- and waterborne diseases listeriosis as a priority to be subjected to enhanced surveillance. In 2008, both sporadic and outbreak-associated cases of infection started to be collected and disseminated through The European Surveillance System. In 2012, ECDC implemented The European Surveillance System with Molecular Surveillance System to routinely collect pulsed-field gel electrophoresis (PFGE) molecular-typing data of *L. monocytogenes* and other foodborne pathogen strains isolated from humans (Acciari et al., 2016). Since 2018 within the European Union/European Economic Area and in Italy in 2019, whole-genome sequencing (WGS) has replaced PFGE for typing and cluster analyses of *L. monocytogenes* cases of infection, using the core genome multilocus sequence type scheme established by Moura et al. (2016). ECDC aimed to evaluate the effectiveness of WGS in a routine epidemiological surveillance system that promoted a large-scale, retrospective, multicenter study on *L. monocytogenes* strains isolated in human case sequences from European Union/European Economic Area countries from 2010 to 2016. Italy participated in the study, with sequences of 132 *L. monocytogenes* clinical strains selected by geographic distribution and PFGE profiles (Van Walle et al., 2018). In the present study, the complete sequences of 132 clinical strains were used to define the evolutionary relatedness among subtypes of *L. monocytogenes* with the aim of (a) identifying clades or clusters more often associated with outbreaks or sporadic cases (Jackson et al., 2016; Moura et al., 2016) and (b) investigating on the persistence of particular strains isolated in different Italian regions from 2010 to 2016.

## Materials and Methods

### Bacterial Strains

During the period 2010–2016, the Istituto Superiore di Sanità collected 826 *L. monocytogenes* clinical strains from patients with invasive listeriosis from different Italian regions, including from the north (82.2%), center (16%), and south (1.8%) of the country. For this study, 132 *L. monocytogenes* strains that were selected both on the geographical distribution and serological and PFGE profiles (Van Walle et al., 2018) were subjected to WGS. Sequencing data were deposited in European Nucleotide Archive (ENA) at http://www.ebi.ac.uk/ena/browser/view/PRJEB45702.

### DNA Extraction and Whole-Genome Sequencing

DNA was extracted and purified from overnight bacterial cultures by MasterPure™ Gram Positive DNA purification kit (Lucigen, Epicentre) according to the manufacturer’s instructions. DNA concentration was evaluated by Quantus fluorimeter (Promega, United States), and 2 ng of total DNA was used for library preparation, using Nextera XT DNA kit and sequenced on NextSeq 500 (111 isolates) and MiSeq (20 isolates) (Illumina, Inc., San Diego, CA, United States). High Output Kit v2 with paired-end 150-nt reads (300 cycles) was used for NextSeq 500 and v3 Reagent kit (600 cycles) paired-end for MiSeq following manufacturer’s instructions. Raw reads were mapped to a reference genome, *L. monocytogenes* FSL F2-208 (accession number CM001046), using the Bowtie2 v.2.3.5 followed by the samtools-bcftools-vcfutils pipeline^1^ to extract the variants [single-nucleotide polymorphisms (SNPs)]. Missing data and ambiguous bases were not allowed at any position, and they were removed by filtering. A final alignment of 175,774 SNPs was created.

### Multilocus Sequence Typing

To determine the level of genetic diversity between isolates, sequence types (STs) were determined by MLST using seven housekeeping genes, including ABC transporter (abcZ), beta-glucosidase (bglA), catalase (cat), succinyl-diaminopimelate desuccinylase (dapE), D-amino acid aminotransferase (dat), L-lactate dehydrogenase (ldh), and histidine kinase (IhkA). The contig files, *de novo* assembly, for each of the draft genomes were uploaded to the Center for Genomic Epidemiology MLST 1.8 with *L. monocytogenes* as the MLST scheme (Larsen et al., 2012). The clonal complexes (CCs) and the STs were defined based on the MLST profile of the isolate having matching profiles at six of seven genes (Ragon et al., 2008; Moura et al., 2016).

### Phylogenetic and Evolutionary Analyses

To perform phylogenetic and evolutionary analysis, six datasets were created.

The first dataset included 33 *L. monocytogenes* strains, classified as lineage I with MLST analysis. The second dataset included 99 *L. monocytogenes* strains, classified as lineage II with MLST analysis. These two datasets were used to perform maximum likelihood (ML) trees.

The nucleotide substitution model was chosen according to the Bayesian information criterion for all the datasets. Statistical support for internal branches of the ML tree was evaluated by bootstrapping (1,000 replicates). ML analysis was performed with IQTREE v.1.6.11 (Trifinopoulos et al., 2016) on the first and second datasets.

The other four datasets were built to perform evolutionary analysis, Bayesian dated-tree, and phylogeographic tree. These datasets included: *L. monocytogenes* strains classified as CC1 (third dataset), *L. monocytogenes* strains classified as CC7 (fourth dataset), *L. monocytogenes* strains classified as CC101 (fifth dataset), and *L. monocytogenes* strains classified as CC155 (sixth dataset). Phylogenetic signal was assessed by likelihood mapping using Tree Puzzle (Schmidt et al., 2002). Analysis of the temporal signal and “clock likeness” of molecular phylogenies on the datasets was performed using TempEst v.1.5.3 (Rambau et al., 2016). This analysis was performed to evaluate the robustness in the molecular clock of the third, fourth, fifth, and sixth datasets. Bayesian Markov chain Monte Carlo (MCMC) method, implemented in BEAST v. 1.10.4^2^ (Suchard et al., 2018), was used to estimate the demographic history of *L. monocytogenes* by calibrating a molecular clock. To investigate the demographic history, independent MCMC runs were carried out enforcing both a strict and relaxed clock with an uncorrelated lognormal rate distribution and one of the following coalescent priors: constant population size, exponential growth, non-parametric smooth skyride plot Gaussian Markov random field, and non-parametric Bayesian skyline plot with ascertainment bias correction. Marginal likelihood estimates for each demographic model were obtained using path sampling and steppingstone analyses (Drummond et al., 2005). Uncertainty in the estimates was indicated by 95% highest posterior density (95% HPD) intervals, and the best-fitting model for each dataset was by calculating the Bayes factors (BF) (Suchard et al., 2018). In practice, any two models can be compared to evaluate the strength of evidence against the null hypothesis (H0), defined as the one with the lower marginal likelihood: 2lnBF < 2 indicates no evidence against H0; 2–6, weak evidence; 6–10: strong evidence; and >10 very strong evidence. Chains were conducted for at least 100 × 10^6^ generations and sampled every 10,000 steps for each molecular clock model. The convergence of the MCMC was assessed by calculating the effective sample size for each parameter. Only parameter estimates with effective sample size’s of >250 were accepted. The maximum clade credibility (MCC) tree was obtained from the tree’s posterior distributions after a 10% burn-in, with the Tree-Annotator software v 1.10.4, included in the Beast package. Statistical support for specific monophyletic clades was assessed by calculating the posterior probability (*pp* > 0.90). The continuous-time Markov chain process over discrete sampling locations implemented in BEAST (Suchard et al., 2018) was used for the phylogeography inference using the Bayesian stochastic search variable selection model, which allows the diffusion rates to be zero with a positive prior probability. Locations considered were the different Italian regions. Comparison of the posterior and prior probabilities of the individual rates being zero provided a formal BF for testing the significance of the linkage between locations. The MCC tree with the phylogeographic reconstruction was selected from the posterior tree distribution after a 10% burn-in using the Tree Annotator.

## Results

Table 1 reports the distribution of CCs and the relative STs of the *L. monocytogenes* sequence strains based on MLST. The most frequent CCs were CC7 (35 isolates), CC1 (20 isolates), CC101 (18 isolates), and CC155 (14 isolates). Specifically, it was possible to show that ST7 was prevalent (32 isolates) in the CC7, ST1 (19 isolates) in CC1, ST38 (17 isolates) in CC 101, and all the sequence strains included in CC155 that belonged to ST 155 (14 isolates). Results proved that CC1, CC7, CC101, and CC155 persist over time in Italy, and some of these have spread to more than one region. Particularly, the CC1 seemed to have an epidemic entrance in 2004 in Emilia-Romagna, moving to Lombardia, Marche, and Piemonte in 2006. CC7 is the oldest CC in Italy, with a presumable entrance in 1999 in Lombardia, moving to Emilia-Romagna in 2010. The CC101 appears to have the entrance in Lombardia in 2008 and does not suggest having evident circulation outside the region, although strains that belonged to CC101 were also isolated in Piemonte in 2013 and in Trentino-Alto Adige in 2015.

**TABLE 1:** Distribution of clonal complexes (CCs) and relative sequence types (STs) of *L. monocytogenes* sequences based on MLST.

CCs	*N*	%	STs	*N*	%
CC1	20	15	ST1	19	15
			ST595	1	1
CC2	2	2	ST2	2	2
CC3	1	1	ST3	1	1
CC4	5	4	ST4	4	3
			ST219	1	1
CC5	3	2	ST5	3	2
CC6	2	2	ST6	2	2
CC7	35	27	ST7	32	24
			ST24	1	1
			ST40	1	1
			ST511	1	1
CC8	8	6	ST8	7	5
			ST120	1	1
CC9	2	2	ST9	2	2
CC14	2	2	ST14	2	2
CC26	1	1	ST26	1	1
CC29	7	5	ST29	7	5
CC31	2	2	ST325	2	2
CC37	3	2	ST37	3	2
CC89	2	2	ST391	2	2
CC101	18	14	ST38	17	13
			ST101	1	1
CC121	2	2	ST121	2	2
CC155	14	11	ST155	14	11
CC398	2	2	ST398	2	2
CC451	1	1	ST451	1	1
Total	132	100	Total	132	100

### Phylogenetic Analysis

**FIGURE 1:**
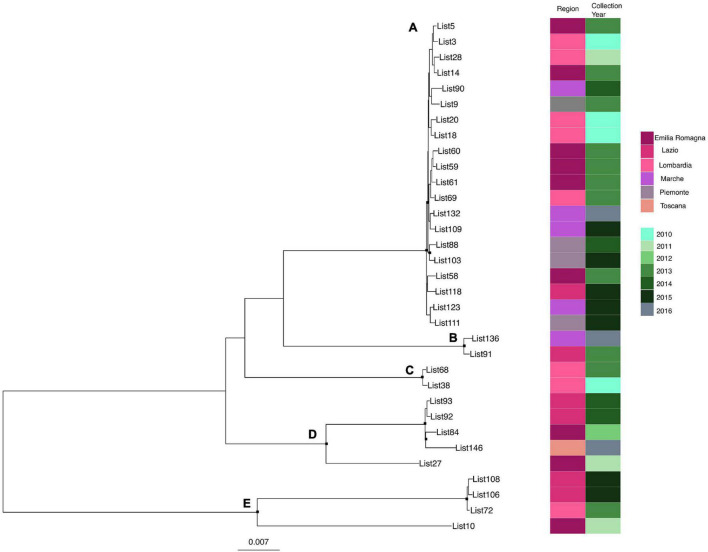
Maximum likelihood of *L. monocytogenes* lineage I strain SNP alignment (first dataset). Significant statistical support (bootstrap values > 0.70%) as indicated by a black rectangle for clade subtending that branch. Clusters are highlighted. Scale bar at bottom of tree indicates 0.007 nucleotide substitutions per site. Heatmap on left showed Italian region membership and collection year for each strain. **(A,D,E)** Clades. **(B,C)** Mean clusters.

**FIGURE 2:**
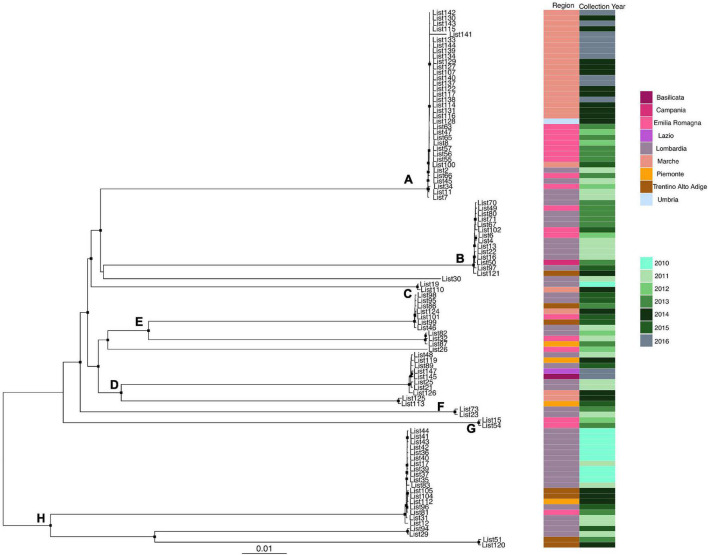
Maximum likelihood of *L. monocytogenes* lineage II strain SNP alignment (second dataset). Significant statistical support (bootstrap values > 0.70%) as indicated by a black rectangle for clade subtending that branch. Clusters are highlighted. Scale bar at bottom of tree indicates 0.01 nucleotide substitutions per site. Heatmap on left showed Italian region membership and collection year for each strain. **(A,B,D,E,H)** Mean clades. **(C,F,G)** Mean clusters.

Likelihood mapping analysis indicated star-like signal (phylogenetic noise) under 6.8% for all datasets; this signified that enough signal for phylogenetic inference was present. The evolutionary model of substitution chosen according to the Bayesian information criterion was GTR + F + I for both the first and second datasets and HKY + G + I for the remaining datasets. Figure 1 shows the ML of the *L. monocytogenes* lineage I strain SNP alignment (first dataset). There have been highlighted three clades (A–D–E) and two clusters (B–C) that were statistically supported (bootstrap values > 0.7). Clade A included the CC1 *L. monocytogenes* intermingled strains isolated from 2010 to 2015, forming different clusters (Lombardia, Emilia-Romagna, Marche, Piemonte, and Lazio); clade D included CC4 *L. monocytogenes* strains isolated from 2011 to 2016 forming different clusters (Emilia-Romagna, Toscana, and Lazio); clade E included different clusters: one *L. monocytogenes* strain belonged to ST3 isolated in 2011 in Emilia-Romagna, and three *L. monocytogenes* strains ST5 isolated in Lazio and Lombardia in 2013 and 2015. Cluster B included the two ST2 *L. monocytogenes* strains isolated in Marche and Lazio in 2013 and 2016; cluster C included two *L. monocytogenes* strains that belonged to ST6 isolated in Lombardia in 2010 and 2013. Figure 2 shows the ML of the *L. monocytogenes* lineage II strain SNP alignment (second dataset). There were five clades (A–B–D–E–H) and three clusters (C–F–G), all statistically supported (bootstrap value > 0.7). With regard to clades, clade A included the *L. monocytogenes* intermingled strains that were classified as CC7 forming different clusters. The main clusters included strains that were mainly isolated in Marche (2015–2016) and in Umbria (2015), Emilia-Romagna, and Lombardia; clade B included *L. monocytogenes* intermingled strains forming different clusters that belonged to CC155 (Trentino, Lombardia, Campania, and Emilia-Romagna); clade D included eight *L. monocytogenes* intermingled strains that form different clusters that belonged to CC8 and two ST9 strains; clade E included three ST37 *L. monocytogenes* strains and seven *L. monocytogenes* strains, classified as ST29, and outside of this cluster, there was as an *L. monocytogenes* strain that belonged to ST26; clade H included two clusters, the ST121 and ST14 groups, with sequences from the same regions each (Lombardia and Trentino) and the CC101 *L. monocytogenes* intermingled strain group (Lombardia, Trentino, Piemonte, and Emilia-Romagna). With regard to clusters, cluster C included two ST398 *L. monocytogenes* strains that were isolated in Lombardia and Marche in 2010 and 2015, respectively; clusters F and G included two *L. monocytogenes* strains each, CC31 and CC89, respectively.

### Bayesian Phylogenetic Analysis

**FIGURE 3:**
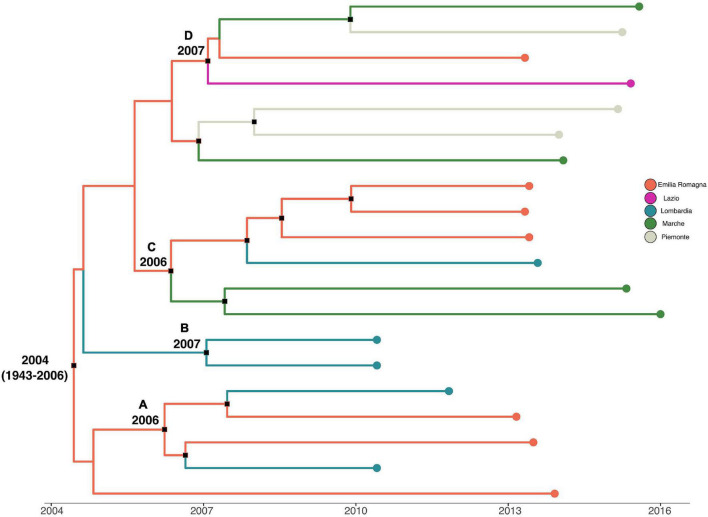
Maximum clade credibility (MCC) tree with Bayesian phylogeographic reconstruction *L. monocytogenes* strain CC1 SNP alignment. Branches are scaled in time and colored according to legend to left, where each color represents geographic location of sampled sequence (tip branches), as well as of ancestral lineage (internal branches) inferred by Bayesian phylogeography. Significant posterior probability support (*pp* ≥ 0.9) as indicated by a black rectangle. Clusters are highlighted.

Analysis of the temporal signal and “clock-likeness” of molecular phylogenies was performed on the third to the sixth dataset. A sufficient correlation between the genetic distance of each sequence to the root of *L. monocytogenes* strain SNP phylogeny and the date of sequence sampling for the datasets analyzed (*r* > 0.54) was found. The exponential growth model with a relaxed clock was selected as the most appropriate to describe the evolutionary history of *L. monocytogenes* strain SNPs’ CC1 and CC155 alignment (lnBF > 6), whereas the Bayesian skyline plot as a demographic model with a relaxed molecular clock was the most appropriate to describe the evolutionary history of *L. monocytogenes* strain SNPs’ CC7 and CC101 alignment (lnBF > 5). MCC tree with a phylogeographic reconstruction of *L. monocytogenes* strain CC1 SNP alignment is shown in Figure 3. The date of the time of the most common recent ancestor (tMRCA) of the root corresponded to 2004 (HPD 95% 1943–2006), probably originated in Emilia-Romagna. Four different statistically supported clusters have been identified (A–B–C–D): cluster A dated back to 2006, probably in Emilia-Romagna, including *L. monocytogenes* strains from Lombardia and Emilia-Romagna from 2010 to 2013; cluster B including two strains from Lombardia in 2010 and it originated in 2007 in Lombardia; cluster C was composed by two subclusters: one including Marche strains from 2015 and 2016 and the other subcluster including 2013 strains from Lombardia and Emilia-Romagna. This cluster dated back to 2006, probably originated in Emilia-Romagna; cluster D dated back to 2007 in Emilia-Romagna and included strains from Emilia-Romagna in 2013, Piemonte, Lazio, and Marche in 2015.

**FIGURE 4:**
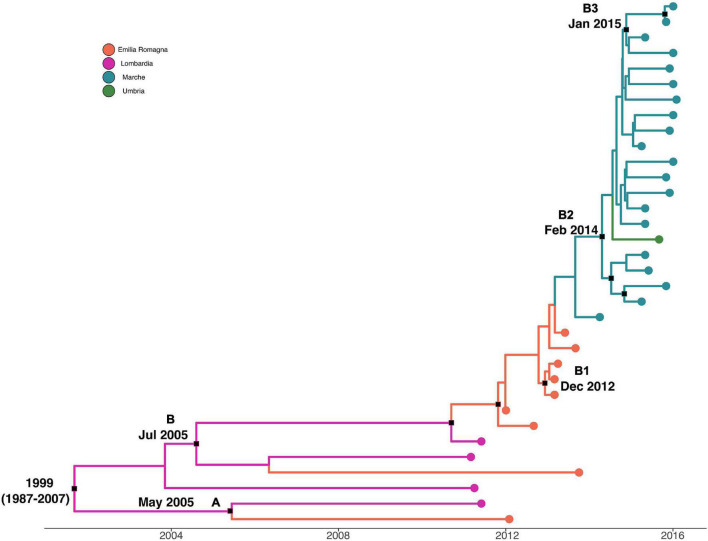
Maximum clade credibility (MCC) tree with Bayesian phylogeography reconstruction *L. monocytogenes* strain CC7 SNP alignment. Branches are scaled in time and colored according to legend to right, where each color represents geographic location of sampled sequence (tip branches), as well as of ancestral lineage (internal branches) inferred by Bayesian phylogeography. Significant posterior probability support (*pp* ≥ 0.9) as indicated by a black rectangle. Clusters are highlighted.

Maximum clade credibility tree with a phylogeographic reconstruction of *L. monocytogenes* strain CC7 SNP alignment is shown in Figure 4. The date of tMRCA of the root corresponded to 1999 (HPD 95% 1987–2007), probably originated in Lombardia. Two different statistically supported clades (A, B) have been identified: clade A dated back to May 2005, probably originated from Lombardia, which included two strains isolated in 2011 and 2012 from Lombardia and Emilia-Romagna, and clade B dated back to July 2005, probably in Lombardia. Clade B included three clusters statistically supported: cluster B1 included Emilia-Romagna outbreak in 2013, dated back to December 2012; cluster B2 included strains from Marche and Umbria in 2015–2016, dated back to February 2014; and lastly, cluster B3 included strains from Marche isolated in 2015 and 2016, probably originated in January 2015.

**FIGURE 5:**
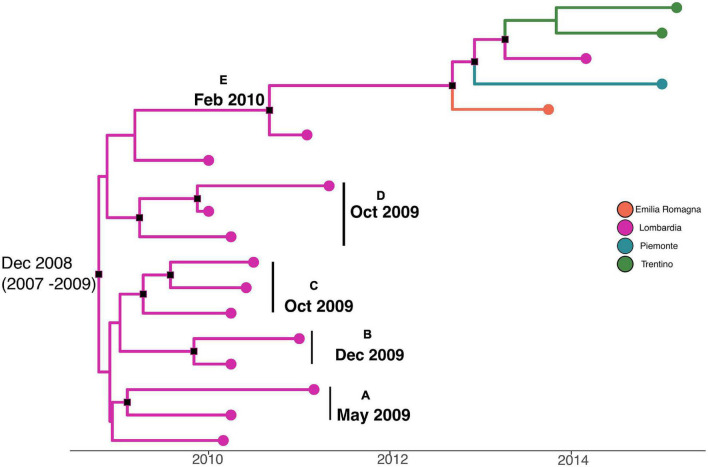
Maximum clade credibility (MCC) tree with Bayesian phylogeography reconstruction *L. monocytogenes* strain CC101 SNP alignment. Branches are scaled in time and colored according to legend to left, where each color represents geographic location of sampled sequence (tip branches), as well as of ancestral lineage (internal branches) inferred by Bayesian phylogeography. Significant posterior probability support (*pp* ≥ 0.9) as indicated by a black rectangle. Clusters are highlighted.

A phylodynamic reconstruction of *L. monocytogenes* strain CC101 SNP alignment is shown in Figure 5. The date of tMRCA of the root is December 2008 (HPD 95% 2007–2009), probably originated in Lombardia. Five statistically supported clusters have been found (A–B–C–D–E), all probably originated in Lombardia: cluster A included strains that originated in May 2009; clusters B, C, and D included strains that originated in December 2009 and October 2009 (for cluster D), and cluster E dated back to February 2010. The latter cluster included strains from Lombardia, Emilia-Romagna, Piemonte, and Trentino that were isolated in different years (2011, 2013, 2014, and 2015).

**FIGURE 6:**
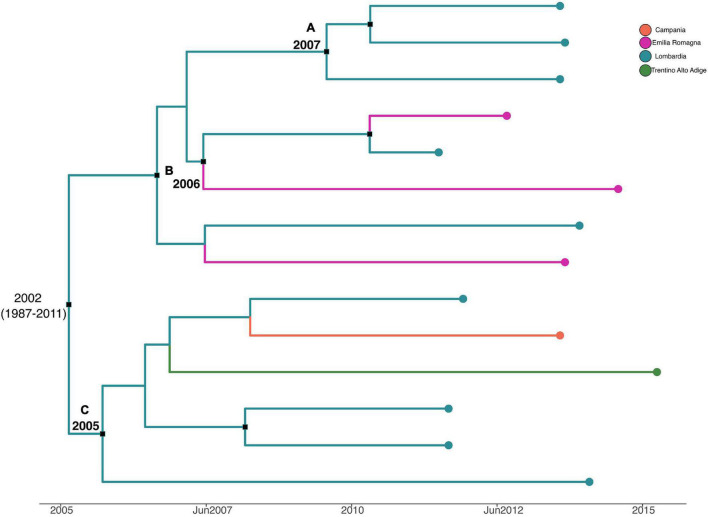
Maximum clade credibility (MCC) tree with Bayesian phylogeography reconstruction *L. monocytogenes* strain CC155 SNP alignment. Branches are scaled in time and colored according to legend to left, where each color represents geographic location of sampled sequence (tip branches), as well as of ancestral lineage (internal branches) inferred by Bayesian phylogeography. Significant posterior probability support (*pp* ≥ 0.9) as indicated by a black rectangle. Clusters are highlighted.

Figure 6 shows the MCC tree with a phylogeographic reconstruction of *L. monocytogenes* strain CC155 SNP alignment. Three different statistically supported clades have been highlighted (A–B–C). The date of tMRCA of the root is 2002 (HPD 95% 1987–2011), probably originated in Lombardia. Specifically, clade A included strains from Lombardia that were isolated in 2013, probably originated in Lombardia in 2007; clade B included two strains isolated in Lombardia and Emilia-Romagna in 2011 and 2012, respectively, and probably originated in Lombardia in 2006, and clade C included six strains that were isolated from Lombardia, Trentino, and Campania, dated back to 2005, which probably originated in Lombardia.

## Discussion

Molecular characterization of the strains has been widely used for analyzing the genetic diversity of the *L. monocytogenes* isolates involved in temporally and geographically unrelated outbreaks to evaluate a communal ancestral strain. Recently, WGS has provided enhanced resolution over traditional subtyping methods and can accurately distinguish isolates that would otherwise be overlooked with traditional subtyping methods. Previous works have demonstrated the ability that WGS has to distinguish between *L. monocytogenes* strains and provide robust phylogenetic evidence linking clinical cases (Jackson et al., 2016; Moura et al., 2016). This ability is crucial for outbreaks of listeriosis that are often temporally extended and usually involve small numbers of (apparently) sporadic illnesses. In addition, epidemiological information is often difficult to collect because of the lengthy incubation period and the severity of the illness. On the other hand, WGS combined with epidemiological information has the potential to attribute relatedness among *L. monocytogenes* strains and thus establish stronger links between human listeriosis cases and causative foods (Bergholz et al., 2016). Therefore, timely typing of pathogens is essential to evaluate the real persistence of clusters in determinate geographic regions. In Italy, the majority of the listeriosis cases reported and the correlated strains collected come from Northern Italy, particularly from Lombardia and Emilia-Romagna. Even if this evidence could suggest that there is a strong underreporting/underdiagnosis in the listeriosis cases in the South of Italy (Pontello et al., 2021), it should also be possible to suspect a lower incidence due to different food practices. Recently, a study performed on food and clinical *L. monocytogenes* strains in northern Italy (Lombardia and Piemonte regions) demonstrated that the same clone was persistent for years (2004–2015) in the Gorgonzola processing plants (Filipello et al., 2017). The strains included in this work have been chosen to represent the Italian situation concerning the clinical strains, isolated from patients with invasive listeriosis, in different Italian regions from 2010 to 2016. In this study, for every clade and cluster, probable epidemic entrances were defined for different years and in different regions. All the analyzed strains belonged to lineages I and II, and the majority of the strains were classified as lineage II (75%). Lineage II included also serotype 1/2a that resulted in the more frequently isolated in Italy in clinical cases (Gianfranceschi et al., 2009). Serotype 1/2a was found more frequently than serotype 4b in listeriosis cases and outbreaks occurring in Europe and the United States between 2010 and 2016 and underlining the hypothesis that serotype 1/2a may be better suited to survive and grow in food and food production (Lomonaco et al., 2015), probably due to its high resistance to disinfection procedures (Brauge et al., 2018). This study provides the first view of *L. monocytogenes* clonal diversity in Italy. The majority of the strains are included in four large CCs (CC1, CC7, CC101, and CC155) and appear to prove the presence of persistence of *L. monocytogenes* in Italy. Particularly in lineage I, approximately 60% of the strains (68.9% of the total *L. monocytogenes* serovars 4b) of *L. monocytogenes* belonged to CC1. The CC1 seemed to have an epidemic entrance in 2004 in Emilia-Romagna, moving to Lombardia, Marche, and Piemonte in 2006. CC1 was strongly associated with strains of clinical origin and reported as the most frequent clone isolated from dairy products (Maury et al., 2016, 2019). In lineage II, the majority of the strains of *L. monocytogenes* sequenced belonged to three main CCs (CC7, CC101, and CC155). In particular, the analysis of the temporal signal and “clock-likeness” of molecular phylogenies established different epidemic entrances of different CCs of lineage II. CC7 is the oldest CC in Italy, with a presumable entrance in 1999 in Lombardia, moving to Emilia-Romagna in 2010. The same CC caused a large outbreak in Marche in 2015 (Duranti et al., 2018). CC155 seems to have its entrance in Lombardia in 2002, where it is apparently limited. CC101 appears to have the entrance in Lombardia in 2008 and does not suggest having evident circulation outside the region, although strains that belonged to CC101 were also isolated in Piemonte in 2013 and in Trentino-Alto Adige in 2015. Among the most frequent CCs isolated in Italy during the period 2010–2016, CC1, CC101, and CC155 are widespread and linked to listeriosis cases in the world, as reported in several studies (Chenal-Francisque et al., 2011; Huang et al., 2015; Maury et al., 2016; Kuch et al., 2018; Zhang et al., 2020). Particularly, the CC1 is the most prevalent genotype in Europe and America (Chenal-Francisque et al., 2011), and CC7 isolates were globally recovered in North and South Americas, Europe, Oceania, Africa, and Asia, from a variety of sources and human infections (Kim et al., 2018). Moreover, a recent study showed that CC1 and CC7 represented the most frequent and widespread clones in food-producing plants and retail stores in central Italy (Centorotola et al., 2021).

Our results highlight that WGS is valuable in epidemiological and microbiological surveillance of *L. monocytogenes* in Italy, allowing, also, the monitoring of the pathogen dissemination.

## Conclusion

According to the European annual epidemiological report on sources of zoonoses, zoonotic agents, and foodborne outbreaks, *L. monocytogenes* is one of the main causes of hospitalization and death in Europe (EFSA and ECDC, 2021). Most human listeriosis cases appear to be related to the consumption of ready-to-eat foods contaminated with *L. monocytogenes* (Chlebicz and Ślizewska, 2018; Ricci et al., 2018). The persistence of specific CC strains in food-processing plants for many years, resulting in intermittent food contamination, has been suggested as the probable cause of many outbreaks (Lomonaco et al., 2015). In this study, the prolonged persistence of specific *L. monocytogenes* CCs was found, indicating that some strains are able to survive better than others in food production environments for extended periods. *L. monocytogenes* exploits different mechanisms of adapting to adverse conditions, such as the capacity to form biofilm or to resist the cleaning and disinfection procedures normally applied in food processing plans (Lundén et al., 2003; Manso et al., 2019). As the persistence of *L. monocytogenes* in food-processing environments is still considered the major source of ready-to-eat food contamination, the identification of these strains has to be considered as an integral part of the risk assessment for improving the hazard characterization of *L. monocytogenes* (Koutsoumanis et al., 2019). Phylogenetic and evolutionary genomic analysis using WGS data has demonstrated an ability to identify the persistence of specific strains in humans, the environment, and foods.

## Data Availability Statement

The data presented in the study are deposited in the European Nucleotide Archive (ENA) repository, accession number PRJEB45702. Further inquiries can be directed to the corresponding author.

## Author Contributions

AF, AG, SF, EC, MO, and FL contributed to the experimental work. EC, SA, and MC contributed to the data analysis. MC, AF, and DD contributed to the writing of the final manuscript. MO performed uploading sequencing data in ENA. All authors contributed significantly to the research, and read and agreed to the published version of the manuscript.

## Conflict of Interest

The authors declare that the research was conducted in the absence of any commercial or financial relationships that could be construed as a potential conflict of interest.

## Publisher’s Note

All claims expressed in this article are solely those of the authors and do not necessarily represent those of their affiliated organizations, or those of the publisher, the editors and the reviewers. Any product that may be evaluated in this article, or claim that may be made by its manufacturer, is not guaranteed or endorsed by the publisher.

## Footnotes

1
https://github.com/samtools

2
http://beast.bio.ed.ac.uk
